# Patient‐physician interactions in hereditary angioedema—Key learnings from the coronavirus disease 2019 pandemic

**DOI:** 10.1002/clt2.12300

**Published:** 2023-09-09

**Authors:** Marcus Maurer, Thomas Buttgereit, Markus Magerl, Kathrin Schön, Zsusanna Balla, Henriette Farkas

**Affiliations:** ^1^ Angioedema Center of Reference and Excellence (ACARE) Institute of Allergology Charité – Universitätsmedizin, corporate member of Freie Universität Berlin and Humboldt‐Universität zu Berlin Berlin Germany; ^2^ Allergology and Immunology Fraunhofer Institute for Translational Medicine and Pharmacology ITMP Berlin Germany; ^3^ HAE Patient Wiesbaden Germany; ^4^ Hungarian Angioedema Center of Reference and Excellence (ACARE) Department of Internal Medicine and Haematology Semmelweis University Budapest Hungary; ^5^ HNO‐Praxis Schaffhausen Schaffhausen Switzerland

**Keywords:** COVID‐19 pandemic, disease control, hereditary angioedema, relationship, shared decision‐making

## Abstract

**Background:**

The coronavirus disease pandemic and its containing measures have caused concerns for patients with hereditary angioedema (HAE) and their treating physicians. Both faced challenges surrounding interaction, and communication had to adapt to facilitate appropriate management. Specifically, the pandemic resulted in reduced in‐person contact in clinics. Where possible, telemedicine appointments were offered and treatment outside the hospital setting was encouraged.

**Body:**

The pandemic markedly affected patient‐physician communication, which is essential to maintain partnerships and optimize care. Although patients with HAE are often experts in their condition, guidance by their physicians is essential, especially with the recent shift toward patient‐centered management for rare diseases and shared decision‐making (SDM). SDM enables patients to take control of their disease and allows the risks and benefits of treatment to be discussed with their physicians. This review explores perspectives from patients and physicians in the HAE clinical setting, particularly regarding their experiences with communication throughout the pandemic. We discuss the importance of SDM in rare diseases such as HAE, factors that impact effective communication, and potential solutions.

**Conclusion:**

Since patient‐centered care and SDM have particular relevance in rare diseases in general, we believe our findings could be transferrable and applicable in the management of other rare diseases.

## 1 INTRODUCTION

Hereditary angioedema (HAE) is a rare autosomal dominant disorder. In most cases, mutation of the *SERPING1* gene results in either deficient or defective C1 inhibitor (C1INH), referred to as HAE‐C1INH type I and II, respectively.^1^ C1INH is a plasma serine protease inhibitor that plays an important role in the regulation of multiple systems, including the complement, fibrinolytic, intrinsic coagulation (contact) and most notably, the kinin‐kallikrein system.^2^ Thus, a lack of C1INH in HAE results in increased bradykinin production, enhanced vascular permeability and the development of angioedema.^3^ Less‐frequent forms of HAE with normal C1INH levels have also been identified, caused by mutations in several different genes involved in the contact‐kallikrein‐kinin system and endothelial permeability.^4^, ^5^

Rare diseases are defined as any life‐limiting or chronically debilitating disease affecting <1 person in 2000^6^; the estimated prevalence of HAE is 1:50,000.^1^ The last decade has seen great advances in rare disease identification and management, driven by both patient voice and an EU directive (2009) that required all member states to have a rare disease plan by 2013.^6^ However, patients with HAE or other rare diseases can experience unique challenges, including a lack of information/expertise availability, and potentially a lack of treatment options.^7^

Patients with HAE suffer from recurring, unpredictable swelling attacks predominantly manifesting in the abdomen and on the skin (including hands, feet, face, and genitals).^8^ Laryngeal and tongue swellings as well as attacks affecting muscles, joints, urinary bladder, kidneys, and other body parts have also been reported.^8^ The disabling, disfiguring, painful and debilitating nature of some attacks can negatively impact quality of life (QoL), due to factors such as work or school absence and avoidance of social events.^9^ Alongside the physical burden of HAE attacks, patients with HAE also experience psychological burdens. The unpredictability of attacks, particularly the risk of life‐threatening laryngeal attacks, represents a source of anxiety for patients with HAE.^10^

The diagnosis of HAE is often delayed, and misdiagnoses are common, as symptoms can be similar to those of more common conditions. A recent study from Germany reported misdiagnosis in more than 50% of patients, the most frequent being appendicitis and allergy, and a median diagnostic delay of 15 years.^11^ Laryngeal attacks, although infrequent, are experienced by around half of all patients with HAE,^12^ and represent a mortality risk in undiagnosed/untreated patients,^13^, ^14^ highlighting the importance of a timely diagnosis.

Since HAE is a lifelong and complex disease, the international World Allergy Organization (WAO)/European Academy of Allergy and Clinical Immunology (EAACI) HAE guideline recommends that HAE‐specific, comprehensive, integrated care is available for all patients and that patients are treated by a specialist with expertise in HAE management.^1^ As such, patients with rare diseases are generally managed in specialist centers that have suitable facilities and appropriately trained healthcare professionals (HCPs), such as Angioedema Centers of Reference and Excellence (ACARE).^15^ However, patients often travel to long distances to attend appointments and receive treatment in these centers.^16^ Inconvenience in terms of time expenditure and organization, and the cost of travel to distant centers can be a barrier to health care for patients, especially in rural areas.^17^

Another unique challenge of effective management of HAE is the heterogeneity of disease presentation and burden both between patients and over time in the same patient, necessitating complex care plans.^18^ Patient involvement in the decision‐making process of HAE management is therefore encouraged to develop individual action plans.^1^ The plan should consist of preventive measures and emergency procedures, including instructions for appropriate medication in case of HAE attacks.^18^ Since disease burden, treatment burden, triggers, patient preference, and response to treatment can fluctuate throughout a patient's life, frequent patient‐physician interaction is vital. It is recommended that patients are evaluated regularly, with care plans being adjusted accordingly.^19^, ^20^

As the complement, coagulation, fibrinolytic and kinin‐kallikrein systems have been implicated in coronavirus disease 2019 (COVID‐19) pathogenesis, there were concerns regarding the unknown impact of COVID‐19 on patients with HAE. Data emerged suggesting that the frequency of severe COVID‐19 symptoms was not increased in patients with HAE.^21^ However, studies support the possibility that severe acute respiratory syndrome coronavirus 2 (SARS‐CoV‐2) infection may act as an attack trigger in patients with HAE, although attack severity was not affected.^21^, ^22^ Studies found that restriction measures during the pandemic were associated with an increased number of anxiety‐related HAE attacks,^23^ and patients with HAE experienced increased fear and emotional distress during the pandemic.^24^ There are conflicting data, however, as another study found there to be no significant difference in patients' Angioedema Quality of Life Questionnaire (AE‐QoL) scores or the frequency of HAE attacks in those with SARS‐CoV‐2 infection.^25^

In addition to potential effects on attack frequency and mental health, many challenges arose concerning communication between patients and physicians due to reduced face‐to‐face visits and additional measures to reduce virus transmission.^23^ Both patients and physicians were required to adapt to enable appropriate management of HAE. These solutions may improve HAE patient care and care for other rare diseases beyond the pandemic.

To date, few studies focus on the importance of the patient‐physician relationship in the management of rare diseases, specifically HAE. In this present review, we aim to underline the importance of these interactions, including perspectives from both patients and physicians. Additionally, we will discuss lessons learned during the transformation and adjustment of healthcare infrastructure and patient management paradigms, specifically throughout the COVID‐19 pandemic.

## 2 A PARTNERSHIP BETWEEN PHYSICIANS AND PATIENTS WITH HAE

**FIGURE 1:**
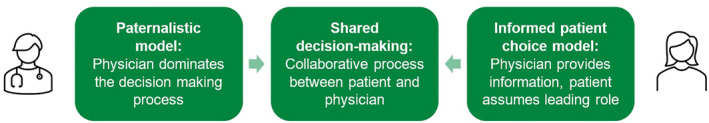
Models of interaction in the treatment and management of patients.

There are several approaches for interaction within the management of diseases, including paternalistic communication, shared decision‐making (SDM), and informed patient choice (Figure 1).^26^ Paternalistic decision‐making by the physician alone occurs often in emergency situations, but may also be important when providing dosages, for example.^26^ The informed patient choice model holds significance within the context of rare diseases, as patients can become experts in their condition throughout their extensive journey in the healthcare system; however, some disadvantages have been noted by physicians, including attempts to self‐diagnose.^26^ SDM is based on cooperation between a patient and a physician to design an individualized care plan reflecting the specific needs and preferences of the patient. The benefits of using SDM in the management of rare diseases include gaining a better understanding of the patient's condition, recognition of the advantages and disadvantages of treatment options, and improved treatment adherence.^26^ A recent study of patient and physician interviews found that patients who participated in high SDM believed that it benefited their care.^27^ The approach used may depend on the level of trust and education within the partnership, the patient's needs in terms of communication style, and the number of treatment options that exist.

A recent consensus by HAE specialists concluded that the ultimate goals of HAE treatment are complete control of the disease and the normalization of the patient's life^28^; a message that was also reflected in the update to the WAO/EAACI guideline for the management of HAE.^1^ Long‐term prophylactic treatment options are currently the only method for potentially achieving these goals.^1^ Since multiple effective treatment options exist for patients with HAE, and because of the fact that ‘normal’ life may look different to each person, patient preference should be considered in the decision‐making process regarding therapy.^29^ Additionally, attack frequency and severity, and perceived impact on quality of life may vary between patients, and throughout an individual patient's life,^19^ necessitating frequent patient‐physician interaction. Further to this, even though QoL assessments such as the Angioedema Activity Score can be useful in gauging whether treatments have reduced the disease burden, they rarely explore the individual's feelings regarding the treatment itself.^30^ Whilst it has been reported that physicians perceive the treatment burden to be higher than patients do, patients tend to ignore the demanding nature of their treatment and accept the treatment burden due to the benefits of the treatment.^31^, ^32^ Open dialog between physicians and patients allows patients to express their concerns and gain advice on how to overcome treatment burdens. A survey reported that 86% of patients would be open to discussion about alternative treatment options that are easier to administer, despite being satisfied with their current treatment.^31^, ^32^ As such, the international guideline recommends that patients should be evaluated for long‐term prophylaxis at every consultation and that treatment plans should be reviewed at least annually.^1^ To aid the use of SDM in HAE management, a three‐stage process termed the ‘3D’ model has recently been proposed: Discover, Discuss and Decide.^33^ The Discover stage aims to identify the patient's needs and preferences and describe treatment options. The Discuss phase includes a discussion between a physician and a patient about how each treatment option aligns with the patient's personal requirements. In the Decide stage, an informed decision on a treatment plan is made together. The decision acknowledges both the advantages and disadvantages of the selected choice, reflects the patient's values and is supported by the previous discussion.^33^ Similar steps were also found to be associated with a high‐SDM approach in a recent analysis by Riedl et al.^27^

Despite the WAO/EAACI guideline recommending the use of SDM when developing a treatment plan for patients with HAE,^1^ reports suggest that the use of SDM in HAE may be variable^27^ and that SDM is not commonly used in rare diseases.^26^ Regardless of recent advances, there are still unmet needs regarding SDM in HAE. The variability in its use is suggestive of a lack of integration into health systems, highlighting the need for education and training for clinicians that SDM is not burdensome and is of benefit to their practice.^29^ Decision aids providing information, benefits, and risks of treatment options in HAE are also lacking for patients, and are required to achieve widespread implementation of SDM.^29^ Finally, perhaps the most important barrier to achieving successful SDM is the ability and time required to build clinician‐patient rapport^29^—a factor that was undoubtedly affected by the COVID‐19 pandemic.

Key factors to consider for SDM are patient‐reported outcome measures (PROMs). PROMs are useful tools that focus on daily health and function from the patient's perspective. Given the sporadic nature of HAE attacks, PROMs are helpful tools to monitor disease symptoms and treatment adherence, and manage disease activity, impact and control. PROMs can also enhance patient‐physician communication to help tailor treatments to meet patients' needs. Consequently, PROM integration in SDM can help to increase patient satisfaction and improve outcomes.^34^

## 3 CHALLENGES AND SOLUTIONS IN COMMUNICATION DURING THE COVID‐19 PANDEMIC

### 3.1 The physicians' perspective

The COVID‐19 pandemic required HCPs to adapt their ways of working, and many were redeployed to intensive care and COVID‐19 wards.^35^ The depleted healthcare resources, in addition to measures adopted to reduce viral transmission, such as public lockdowns, social distancing, and travel restrictions, disrupted regular care for patients with rare diseases, including HAE.^36^ A recent international survey asked 61 HAE‐treating HCPs from 36 countries about their perceptions of the current and future impacts of COVID‐19 on HAE management. Of the respondents, 95% believed their practice would be limited in the next 5 years as a result of the pandemic, and 78% expected a permanent change; a greater impact was anticipated by respondents from low‐income countries.^36^ In the same survey, however, only 27% of HCPs were dissatisfied with the services they were able to offer patients with HAE during the pandemic.^36^

Integrated care is vital for HAE patients, with interactions and collaboration among physicians required at many stages throughout a patient's journey with the condition. Communication between HAE specialist centers and general practitioners (GPs) is necessary to ensure the recommended screening of family members, including children and grand‐children of a patient diagnosed with HAE.^1^ According to the experience of the authors at the Hungarian ACARE center, during the COVID‐19 pandemic, several processes were implemented to support the patient journey, without necessarily requiring visits to HAE specialist centers in hospitals. Firstly, physicians used ‘request for consultation’ forms—a questionnaire that contained targeted questions and provided pictures of edematous attacks to HAE specialists. Secondly, HAE specialist centers provided local practices with protocols for the preparation, storage, and transfer of blood samples that were collected for biochemical and genetic diagnostic testing. Thirdly, HAE‐specialist physicians asked local physicians to educate patients about self‐administration and perform ultrasounds or routine check‐ups locally. Lastly, local hospitals stored acute treatment for HAE attacks, which required good working relations and interactions between HAE‐treating physicians, pharmaceutical companies, and pharmacists to ensure continuous treatment supply.

Regarding the interaction with patients, reduced face‐to‐face consultations were common during the pandemic. At the Charité ACARE in Berlin, in‐person consultations were reduced by approximately 60%. Accordingly, the use of telephone/email/video calls increased by approximately 300% (unpublished data). Although the majority of HCPs agree that face‐to‐face consultations are important in the management of HAE, 78% rated online consultations as acceptable, good, or very good as a primary means of managing patients with HAE. In addition, 50% of respondents indicated that they may continue providing telemedicine in the future.^36^ However, communication without face‐to‐face contact can result in several challenges in a patient‐physician relationship. Personal experiences from the current authorship include difficulties in obtaining former diagnostic reports from other physicians, differing requirements or preferences for telephone and/or video calls by individual patients, and volume or connectivity issues. Furthermore, in the absence of in‐person assessments, physicians may not recognize non‐verbal cues such as gestures and body posture.

Early diagnosis and effective therapy are critical for rare diseases such as HAE.^1^ Throughout the pandemic, processes to facilitate diagnosis required adaptation. At the Hungarian ACARE (Department of Internal Medicine and Haematology), the patient's medical history was obtained during a video call. In addition, patients were offered a separate appointment to provide a blood sample during off‐peak hours to reduce the potential exposure to SARS‐CoV‐2. Alternatively, to avoid long‐distance travel, patients were also able to provide a blood sample at their GP, who could send samples to the testing laboratory (Henriette Farkas, personal communication). Dried blood spot testing is currently being investigated in some ACAREs, which could further facilitate and expedite diagnostic testing (physician authors, personal communication). Following diagnosis, the authors report additional considerations such as the appropriateness of delivering bad news over the phone or via video call. There was a risk that reduced face‐to‐face contact could lead to less opportunity for disease education and reluctance to ask questions. A major concern during follow‐up consultations was reports of difficulties using PROMs, as the availability of online versions is currently limited. An Urticaria Centers of Reference and Excellence (UCARE) network survey reported a 10%–20% reduction in the use of PROMs during the pandemic.^37^

### 3.2 The patients' perspective: A case study

KS is a 29‐year‐old patient with HAE type II living in Wiesbaden, Germany. She experienced her first attack (swelling of the face) at the age of 2 years and was diagnosed in 2008 after a delay of 12 years. She had been receiving on‐demand treatment from the time she was diagnosed until February 2020, when she made the decision to switch to prophylactic therapy. Since her diagnosis, she has had extensive experience communicating with physicians. She notes that face‐to‐face appointments are preferred as direct interactions facilitate trust‐based relationships and a better understanding of each other in terms of voice, gestures, and facial expressions.

**FIGURE 2:**
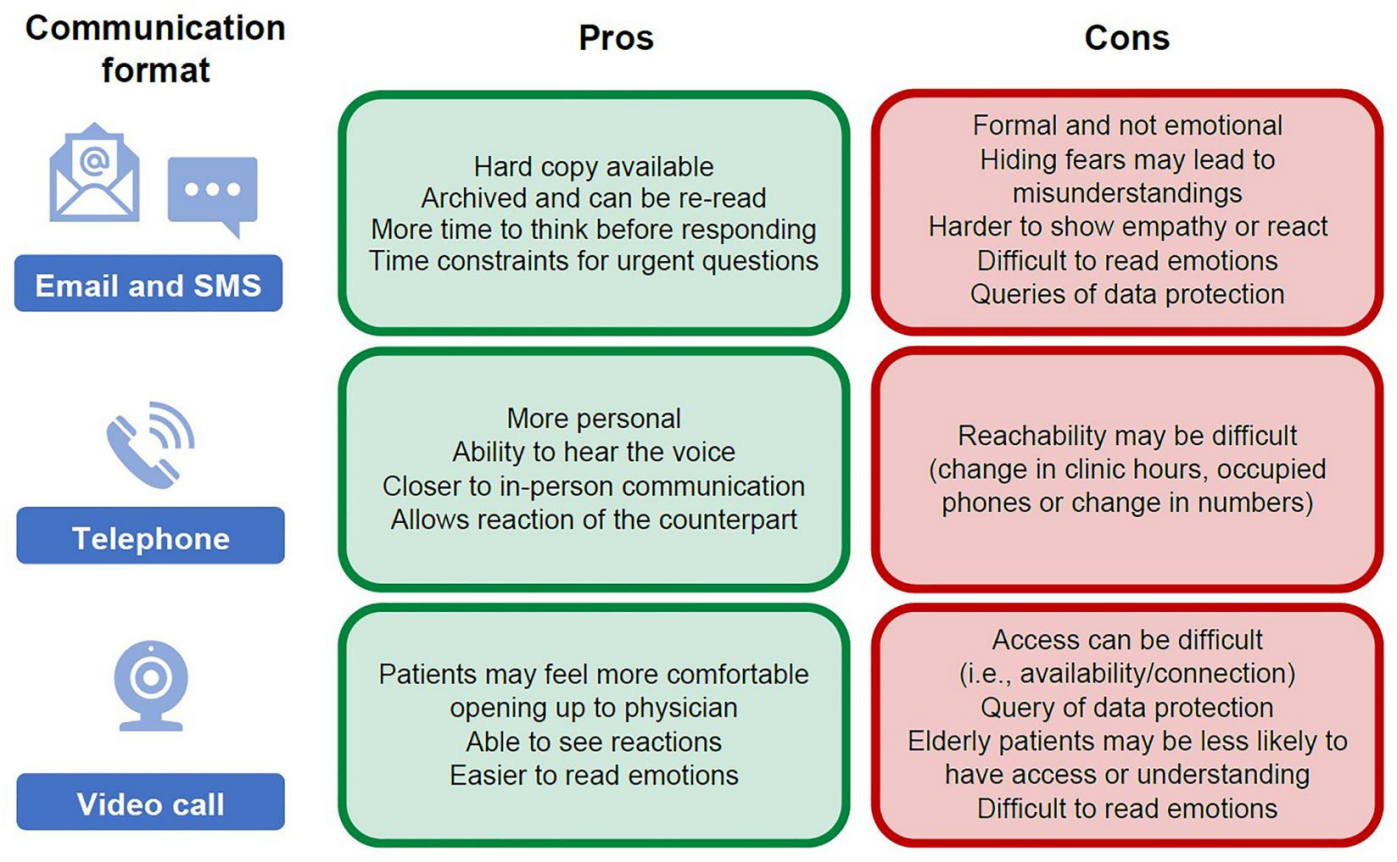
Challenges and solutions in communication with physicians when face‐to‐face consultation is not possible: The patients' perspective.

Moreover, the development of trust in a physician is vital since the disclosure of details about a patient's personal life is often required. In her experience, visits to a medical environment, for example, the hospital, help to normalize these discussions with the physician. When face‐to‐face consultation was not possible during the pandemic, the ability to contact a physician was important for patient reassurance and information regarding possible effects or interactions of COVID‐19 in HAE. A summary of the pros and cons of communication formats other than face‐to‐face in the management of patient care is shown in Figure 2.

**FIGURE 3:**
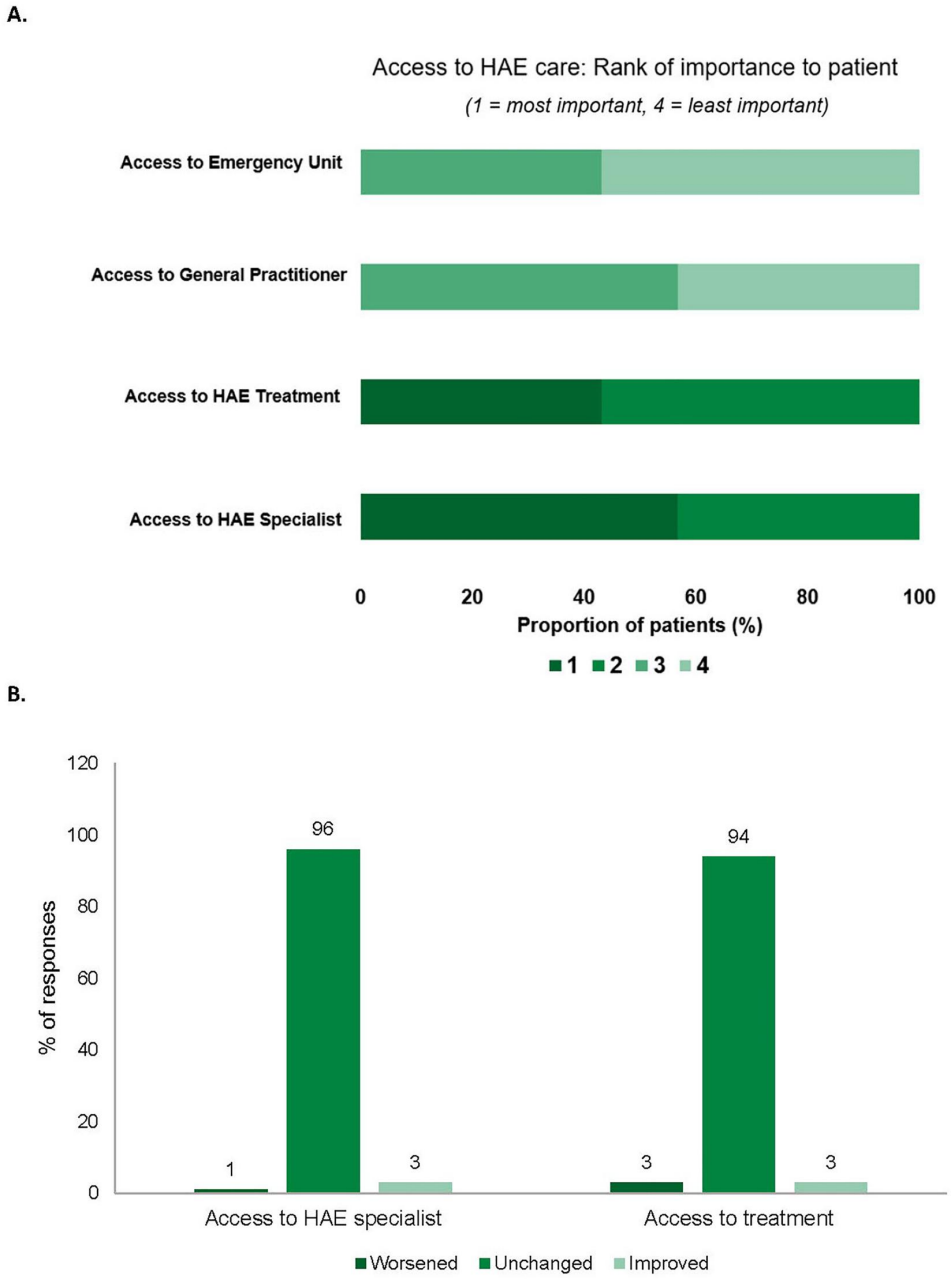
Patient‐ranked importance of access to components of hereditary angioedema (HAE) care (A) and patient‐perceived access to a HAE specialist and treatment (B) during the COVID‐19 pandemic at the Hungarian Angioedema Centers of Reference and Excellence (ACARE).^38^ A telephone interview was completed by 37 randomly selected patients at the Hungarian ACARE in August 2020. Patients ranked the following factors regarding their HAE care in order of importance from 1 (most important) to 4 (least important): access to emergency unit, access to the general practitioner, access to HAE treatment and access to HAE specialist. They were also asked to indicate whether they perceived access to HAE specialists and treatment was worsened, unchanged or improved during the pandemic.

A recent survey from August 2020 was completed by 37 randomly selected patients at the Hungarian ACARE. The survey was conducted via telephone interview, and patients ranked the following factors regarding their HAE care in order of importance from 1 (most important) to 4 (least important): access to emergency unit, access to the general practitioner, access to HAE treatment and access to HAE specialist. Of 37 patients, nearly 60% ranked access to their HAE specialist as the most important factor regarding their care when compared with the other factors (Figure 3A, author's data). The same survey also found that patient‐perceived access to HAE specialists and treatment was unchanged during the pandemic (Figure 3B, author's data^38^).

However, KS notes that during the pandemic, the change in opening hours and the availability of phone operators of centers resulted in challenges in the accessibility and reachability of HCPs. Additionally, while a global increase in the use of telehealth was observed in 2020, its implementation was less successful in lower‐income countries.^36^ Therefore, these patients may have experienced restricted access to HCPs at a crucial time.

It is important to note that this description of the experience of ACARE patients in Hungary and Berlin may be optimistic and not representative of the experiences of patients in all centers, low‐income countries, or with other rare diseases.^36^, ^39^, ^40^ In 2020, a global study, consisting of two parallel surveys, was conducted between two respondent groups: patients with HAE and HCPs (physicians and nurses) who treat patients with HAE. Patient survey responses were collected from 135 people across 31 different countries. Most patients (*n* = 81) resided in high‐income countries (including Israel, the United States of America, Romania etc.). The remaining number of patients (*n* = 54) resided in lower‐income countries (including North Macedonia, Serbia, Turkey etc.). Results from the survey showed that only 49% of patients were satisfied with their care in October 2020 versus 68% pre‐pandemic, and 80% of patients believed that their medical care would be permanently restricted or changed as a result of the pandemic.^36^ More patients from lower‐income countries believed that several aspects of their care will be affected long term relative to those from higher‐income countries, including access to and availability of treatment.^36^

## 4 KEY LEARNINGS AND SOLUTIONS ARISING FROM THE COVID‐19 PANDEMIC

As discussed, the global COVID‐19 pandemic led to drastic changes in the management and treatment of patients across many clinical settings, causing concern for patients and their caregivers or family. Factors perceived by patients to be affected long‐term by COVID‐19 include access to outpatient treatment, scheduled visits, treatment administration, and the availability of both acute and prophylactic treatment. Although it remains to be seen whether this transpires, we believe that the fear that patients have is real, and therefore, HCPs must continue to provide reassurance for patients.

While the value of face‐to‐face appointments cannot be argued, the use of telemedicine as a result of drastic restriction measures has opened up new opportunities that may complement rare disease patient care moving forward. A survey conducted by the National Organization for Rare Disorders involving over 800 patients and caregivers across the US indicated that during the pandemic, the use of telemedicine dramatically increased as a preferred option to face‐to‐face appointments for care.^41^ Over 83% of patients with rare diseases were offered a telehealth visit, and 92% of users described it as a positive experience.^41^ This data surrounding the use of telemedicine is encouraging and may be advantageous for patients who fail to attend healthcare appointments. For example, at the Hungarian ACARE, it was noted that around 10 patients with HAE, who were largely asymptomatic, repeatedly failed to show up for in‐person follow‐up appointments. For these patients, telemedicine may help to increase patient‐physician interactions and SDM to prevent any HAE attacks.

Since its introduction as an option, these patients have indicated that they are willing to be available via telemedicine (Zsusanna Balla, personal communication). Therefore, in some cases, providing telemedicine as an option may increase the frequency of patient‐physician interactions and enable SDM. It must be noted, however, that the use of tele/video communication must be based on individual requirements and resources. For example, a video consultation may be a preferable option to see patients living further away as it enables physicians to gauge patients' emotions; however, this method may not be suitable for elderly patients or those in lower‐income countries.

Alongside tele/video consultations, e‐prescriptions—cloud‐based services available in multiple countries—allow physicians to prescribe medicines online so that patients can obtain their prescriptions directly from the pharmacy without the need for a visit to the clinic (Zsusanna Balla, personal communication). Online tools and apps may facilitate self‐management and supplement SDM in rare diseases such as HAE.^42^ The limited availability of PROMs during the pandemic has spurred research using the UCARE network into the design of online versions that may be useful for chronic urticaria, and potentially HAE, in the future.^37^ However, disparities in resources and global infrastructure need to be addressed to support non‐high‐income countries in case of future events.^36^ Accessible care reassures patients and promotes trust, which forms the basis for a patient‐physician relationship, and should be continued even when face‐to‐face consultations are not possible.

## AUTHOR CONTRIBUTIONS

**Marcus Maurer:** Conceptualization (equal); data curation (equal); formal analysis (equal); investigation (equal); methodology (equal); resources (equal); supervision (equal); validation (equal); visualization (equal); writing – original draft (equal); writing – review and editing (equal). **Thomas Buttgereit:** Conceptualization (equal); data curation (equal); formal analysis (equal); investigation (equal); methodology (equal); resources (equal); supervision (equal); validation (equal); visualization (equal); writing – original draft (equal); writing – review and editing (equal). **Markus Magerl:** Conceptualization (equal); data curation (equal); formal analysis (equal); investigation (equal); methodology (equal); resources (equal); supervision (equal); validation (equal); visualization (equal); writing – original draft (equal); writing – review and editing (equal). **Kathrin Schön:** Conceptualization (equal); data curation (equal); formal analysis (equal); investigation (equal); methodology (equal); resources (equal); supervision (equal); validation (equal); visualization (equal); writing – original draft (equal); writing – review and editing (equal). **Zsusanna Balla:** Conceptualization (equal); data curation (equal); formal analysis (equal); investigation (equal); methodology (equal); resources (equal); supervision (equal); validation (equal); visualization (equal); writing – original draft (equal); writing – review and editing (equal). **Henriette Farkas:** Conceptualization (equal); data curation (equal); formal analysis (equal); investigation (equal); methodology (equal); resources (equal); supervision (equal); validation (equal); visualization (equal); writing – original draft (equal); writing – review and editing (equal).

## CONFLICT OF INTEREST STATEMENT

Medical writing assistance was provided by Meridian HealthComms Ltd., funded by CSL Behring. Marcus Maurer is or recently was a speaker and/or advisor for and/or has received research funding from Alnylam, BioCryst, Centogene, CSL Behring, Dyax, Kalvista, Pharming, Pharvaris, and Shire/Takeda. Thomas Buttgereit is or recently was a speaker and/or advisor and/or consultant for BioCryst, CSL Behring, Takeda, Pharming and KalVista. Markus Magerl is or recently was a speaker and/or advisor for BioCryst, CSL Behring, Octapharma, Pharvaris, Takeda and KalVista. Kathrin Schön is or recently was a speaker for BioCryst, CSL Behring and Takeda. Zsusanna Balla is or recently was a speaker and/or has received research funding from CSL Behring, Pharvaris and Takeda. Henriette Farkas is or recently was a speaker and/or advisor for and/or has received research funding from Astria, BioCryst, CSL Behring, Shire/Takeda, Pharming, KalVista, Pharvaris and ONO Pharmaceutical.

## Data Availability

Data sharing is not applicable to this article as no new data were created or analyzed in this study.
